# Single-Wall Carbon Nanohorn Langmuir–Schaefer Films

**DOI:** 10.1021/acs.langmuir.3c01396

**Published:** 2023-08-16

**Authors:** Kamil Kędzierski, Karol Rytel, Bolesław Barszcz, Łukasz Majchrzycki

**Affiliations:** † Institut of Physics, Poznan University of Technology, 60-965 Poznan, Poland; ‡ Institute of Molecular Physics, Polish Academy of Sciences, 60-179 Poznan, Poland; § Center of Advanced Technology, Adam Mickiewicz University, 61-614 Poznan, Poland

## Abstract

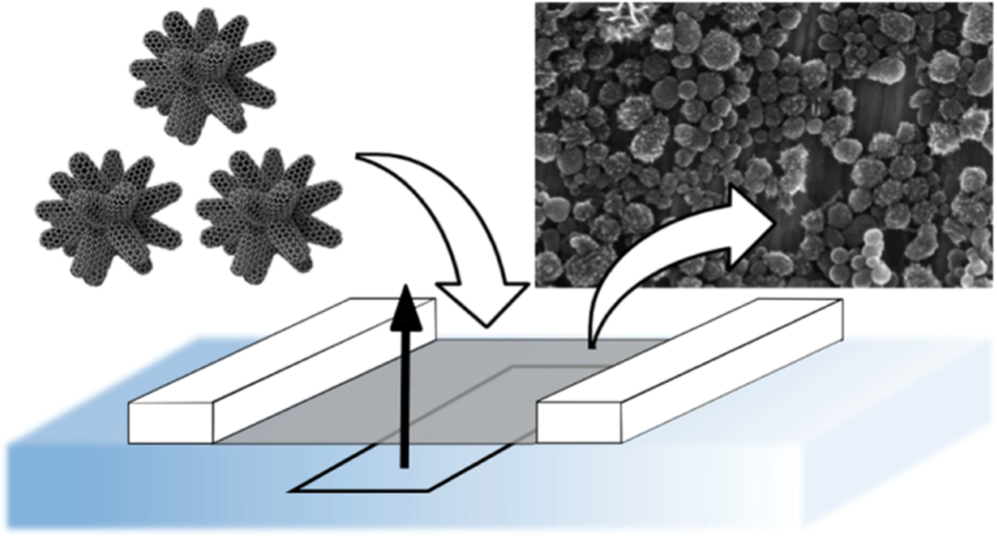

A suspension of single-walled carbon nanohorn (SWCNH) aggregates with a size of approx. 50 nm was used to create a floating film at the water–air interface. The film was then transferred onto large-area quartz substrates using the Langmuir–Schaefer technique at varied surface pressures. The packaging and arrangement of SWCNHs in the film can be controlled during the process. The resulting films’ optical and electrical properties were investigated, and the highest electrical conductivity and figure of merit parameter values were observed for the film transferred at surface pressure near the collapse point. These films had a surface density of less than 5 μg cm^–2^, making them ideal for use in ultra-light sensors, supercapacitors, and photovoltaic cell electrodes. The preparation and properties of the Langmuir–Schaefer films of carbon nanohorns are reported for the first time.

## 1 Introduction

Recently, carbon allotropes in the form of nanostructures have found remarkable interest among scientists all over the world due to their extraordinary properties, easy structure modification, and almost inexhaustible resource of carbon. However, the synthesis of carbon nanomaterials usually requires the use of a catalyst polluting the final product and/or is very time- and energy-consuming. One of the few exceptions is single-wall carbon nanohorns (SWCNHs), which are commonly synthesized via CO_2_ laser ablation of graphite in a controlled atmosphere without the need for a catalyst.^1^ The SWCNHs are tiny graphene sheets, wrapped to form horn-shaped cones of 2–5 nm typical diameters, lengths of 40–50 nm, and cone angles of approx. 20°.^2^ The nanohorns during synthesis create ball-like shape aggregates with diameters of 80–100 nm, yet the aggregates of significantly lower sizes (about 50 nm) were reported.^3^ The SWCNHs were named by Iijima et al.^4^ but similar structures were observed by Harris et al.^5^ Carbon nanohorns stand out from other carbon nanostructures by high active area and pore volume, a possibility for the controlled opening of nanowindows and free movement of molecules encapsulated in the nanohorns interior.^6^ Due to their unique properties, SWCNHs have found possible applications in CH_4_ and CO_2_ detection, Li-ion batteries, hydrogen storage,^7^ and varied forms of sensors.^8^ Moreover, taking into account their low toxicity,^9^ they can be used in pharmacies as drug carriers.^10,11^ Furthermore, the unique electronic structure of SWCNHs leads to the nanohorns’ possible applications as electrode modification in high-power supercapacitors,^12−14^ energy storage devices^15^ dye-sensitized solar cells,^1,16,17^ and field emission devices.^18^

In many applications, to minimalize the size, mass, and cost of final devices, it is desired to create thin films of nanomaterials. One of the best methods of thin-film preparation is the Langmuir technique (L), which allows for the creation of a thin layer or even a monomolecular layer on the water–air interface. Furthermore, the Langmuir layer can be transferred on the solid substrate via horizontal or vertical dipping as so-called Langmuir–Schaefer (LS) and Langmuir–Blodgett methods, respectively. The Langmuir technique allows the creation of a large area of highly organized structures without the need for vacuum conditions.^19^ Despite the L method being designed for small amphiphilic molecules as fatty acid,^20^ it can be easily adapted for other materials such as small molecules,^21^ nanorods,^22^ nanocrystals,^23^ fullerenes,^24^ carbon nanotubes,^19,25−28^ and many others. Kondej et al.^29^ investigated the interactions of SWCNHs with lipids in the model membrane at the water–air interface in relation to health effects from nanoparticle inhalation and potential medical applications of SWCNHs. However, our paper for the first time considers SWCNH Langmuir layers transferred on solid substrates.

Taking advantage of the LS method, we created thin films of the SWCNH aggregates with diameters of ∼50 nm on the solid substrates. The floating film was stable in time and insensible to the compression rate until it exceeded a collapse point. We show that the film package, ultraviolet–visible (UV–vis) transmittance, and electrical resistance can be easily controlled by the surface pressure during the layer transfer. The SWCNH semi-transparent film of specified light transmittance can be suitable for the scaffold of photoelectrochemical sensors.^30,31^ We focus our investigations on films created in one deposition process to reveal the basic properties of the SWCNH single layer. Therefore, the paper considers preliminary research on carbon nanohorn Langmuir–Schaefer layers for possible applications in thin-film scaffolds for sensing devices.

## 2 Methods

In this work, thin films of single-wall carbon nanohorns produced by Carbonium Srl by a process based on rapid condensation of carbon atoms without any catalyst were investigated. The individual horn diameter was between 3 and 5 nm, and the active surface of bulk SWCNHs was about 300 m^2^ g^–1^ and, after mild oxidizing treatment, reached a value of 1600 m^2^ g^–1^ (manufacturer information). An SWCNH suspension was prepared by adding 15 mg of the nanohorn powder to 200 mL of spectrally pure dichloromethane (DCM). Then, the sample was sonicated in an ultrasonic bath (POLSONIC; 300 W) for 30 min, which resulted in suspension stability in time. The SWCNH thin films were obtained by using a 2000 Langmuir–Blodgett system (KSV instruments Ltd.) with a PTFE trough of 750 cm^2^ area (15 cm × 50 cm), under laminar flow at a stabilized temperature of 20 ± 0.5 °C. As a subphase, deionized water (electrical resistivity of 18.2 MΩ·cm) prepared with an ultrapure water purification system (Millipore Co.) was used. For floating film preparation, 7.5 mL of the suspension was carefully spread onto the subphase and DCM was allowed to evaporate for 30 min. Then, the film was compressed symmetrically from both sides at a stable surface compression rate change (***v***), and surface pressure (**Π**) was recorded via the platinum Wilhelmy plate method. The floating films were transferred onto quartz substrates (76 mm × 26 mm) by horizontal transfer, carried out by removal of the substrate from the subphase, in a similar way as in previous studies.^32−35^ Before transfer, the floating films were relaxed at specified **Π** (10, 15, 20, 25, 30, 33, and 35 mN m^–1^) for 15 min to obtain a uniform layer.

UV–vis transmittance spectra in the range of 190–900 nm were recorded using a Cary 4000 spectrophotometer (Varian), and the UV–vis measurements were repeated several times at different places of the samples and no difference was observed. The infrared transmittance spectra were recorded with a Fourier transform infrared (FT-IR) Bruker Equinox 55 spectrophotometer. The samples were prepared by grinding the SWCNH powder in an agate mortar and mixing them with spectroscopic grade potassium bromide (concentration ∼1:2000). The pellets were made from the powder using a simple press, and a pure KBr pellet was used as a reference. The Raman scattering spectra were collected with a Horiba Jobin Yvon LabRam HR800 spectrometer using excitation λ_ex_ = 633 nm. Electrical resistance measurements were performed using a standard two-probe method via a 2400-LV sourcemeter (Keithley). The contacts were made of a silver wire (diameter 0.25 mm), and they were attached to the sample using silver paint at a distance of about 10 mm. The specific distances and confocal microscopy images were obtained via a laser scanning microscope LSM710 (Zeiss) operating at a 543 nm laser beam in material mode. The SEM images were recorded using a Helios NanoLab 660 (FEI) scanning electron microscope operating at 5 kV.

## 3 Results and Discussion

### 3.1 Material Characterization

**Figure 1:**
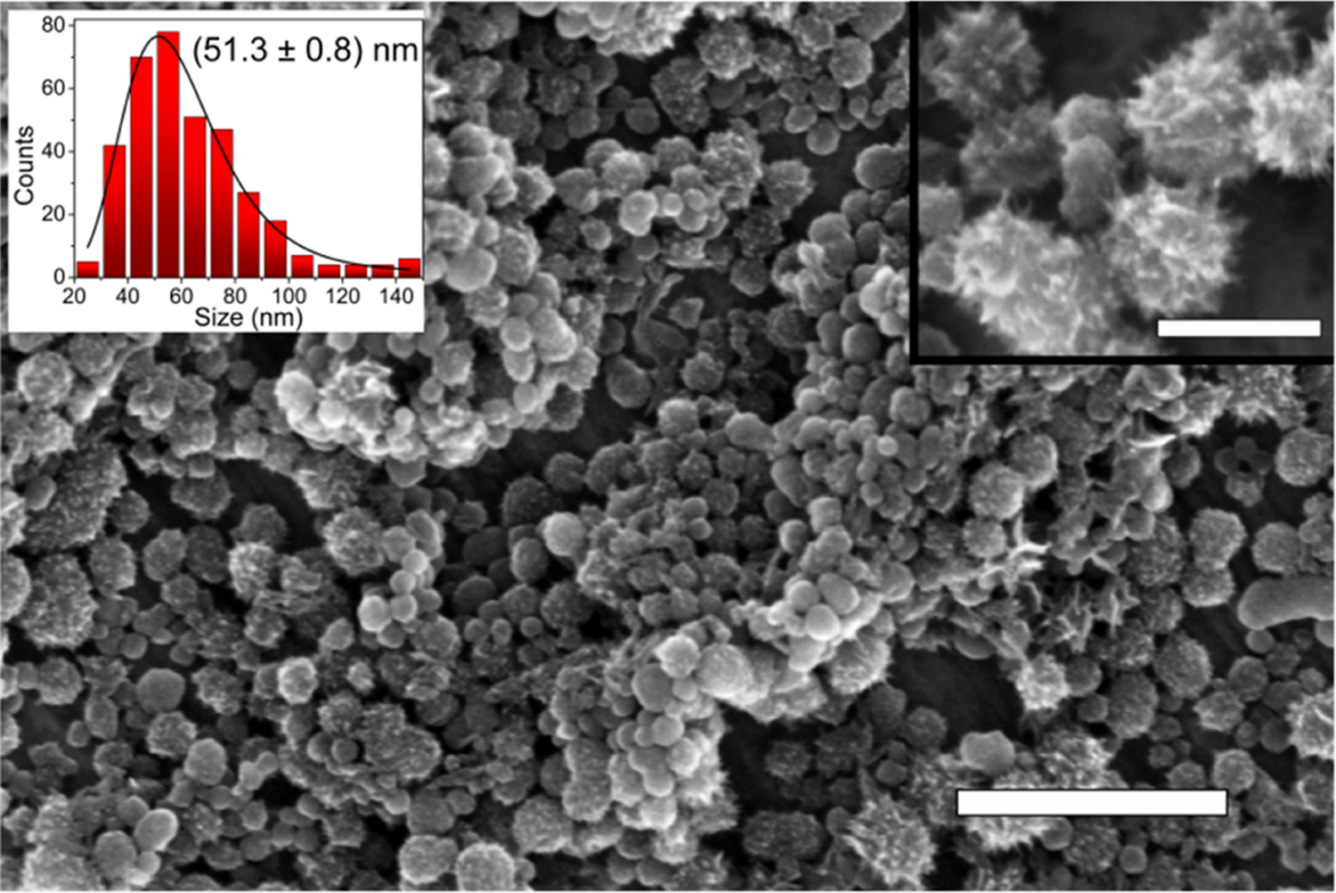
SEM image of SWCNH powder after redrying from DCM suspension. Right inset: higher resolution SEM image of SWCNHs; left inset: size distribution of SWCNHs. Scale bars in the main image and in the inset are 500 and 150 nm, respectively.

Investigated SWCNH aggregates in spheres with an average diameter of 51.3 nm, a standard deviation of 0.8 nm, and a width of the size histogram fit of 16.7 nm. In scanning electron microscopy (SEM) images (Figure 1), a small fraction of aggregates of significantly larger size, up to 150 nm, are present; however, they are much less common than the structures with diameters of about 50 nm. The structures of smooth surfaces are dominant; nevertheless, the aggregates of the irregular surface with sharp narrows are visible (see Figure 1 inset). In most of the articles, investigations of the SWCNH aggregates with significantly larger diameters, i.e., 80–100 nm, were reported.^2^ However, the research concerning nanohorn aggregates with sizes similar to ours was also published.^3,4,36−38^ In bulk powder, sphere shape aggregates create larger agglomerates well, as shown in Figure 1, which are fully decomposed via ultrasonication in DCM—the resulting suspension was homogeneous and stable in time.

**Figure 2:**
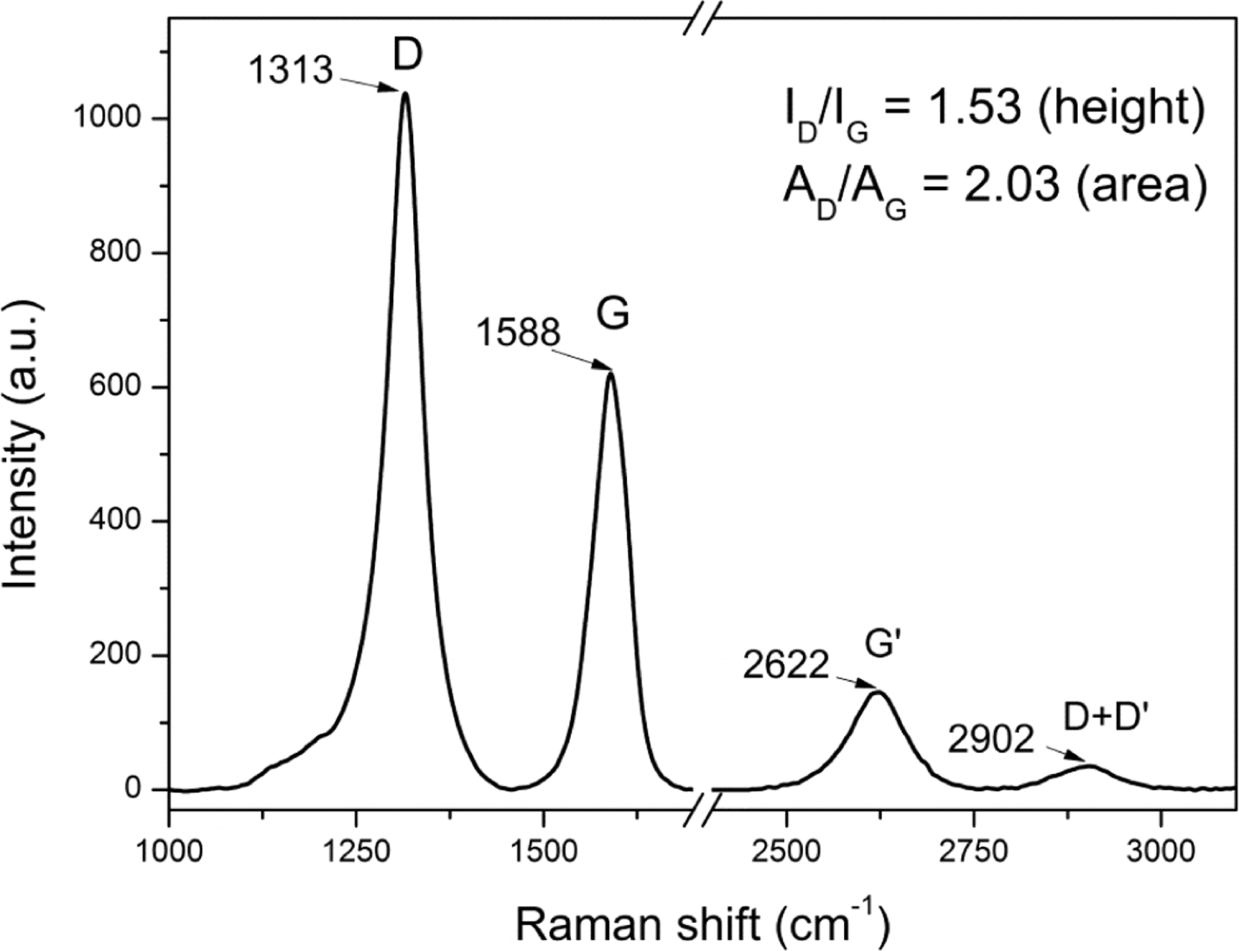
Raman spectrum of SWCNH powder; excitation wavelength = 633 nm; the background was subtracted and the origin spectrum is shown in Figure S1 in the SI.

The Raman spectrum of SWCNHs shows two main bands at 1588 and 1313 cm^–1^ (Figure 2), which can be assigned to G and D modes, respectively.^39^ The G band comes from the intrinsic properties of a graphite sheet. For graphite, the band is observed at 1582 cm^–1^, which is a slightly lower value than for SWCNHs; nevertheless, for carbon nanotubes (CNTs), the G mode is observed at 1590 cm^–1^. A defect-induced D band in SWCNHs results from the two topological distortions: layer folding and pentagon carbon rings in a conical SWCNH structure.^40^ The recorded G mode energy at 1588 cm^–1^ follows literature reports; however, the D band for SWCNHs is usually observed for higher wavenumbers (i.e., 1340–1350 cm^–1^) than that in our case.^41−44^ One must remember that despite other factors, the Raman D band is dependent on the excitation energy (laser wavelength).^45^ The cited research utilized mainly 488, 514, and 532 nm as the Raman excitation source. Taking this into account, the position of the D band at 1313 cm^–1^ for 633 nm excitation in our case is quite typical (see ref (47) for more information about mentioned dependence). Both bands are relatively broad—full widths at half-maximum are about 48 and 64 cm^–1^ for G and D bands, respectively. Furthermore, in the Raman spectrum, two less intense bands at 2622 and 2902 cm^–1^ were observed and assigned as G′ and D + D′ modes, respectively. The G′ band is an overtone of D mode,^39^ and the D′ band originates from disorder and often is present as a shoulder of the G band, but in our case, it is covered. The D to G band intensity ratio value (*I*_D_/*I*_G_) of about 1.5 indicates a highly defected structure of the nanohorn sites but the site defects can be regenerated.^3^ Nevertheless, a highly damaged SWCNH structure with open nanowindows due to SWCNHs’ high active area can be very attractive for applications in storage or sensing devices.^7^

### 3.2 SWCNH Film Morphology/Arrangement

**Figure 3:**
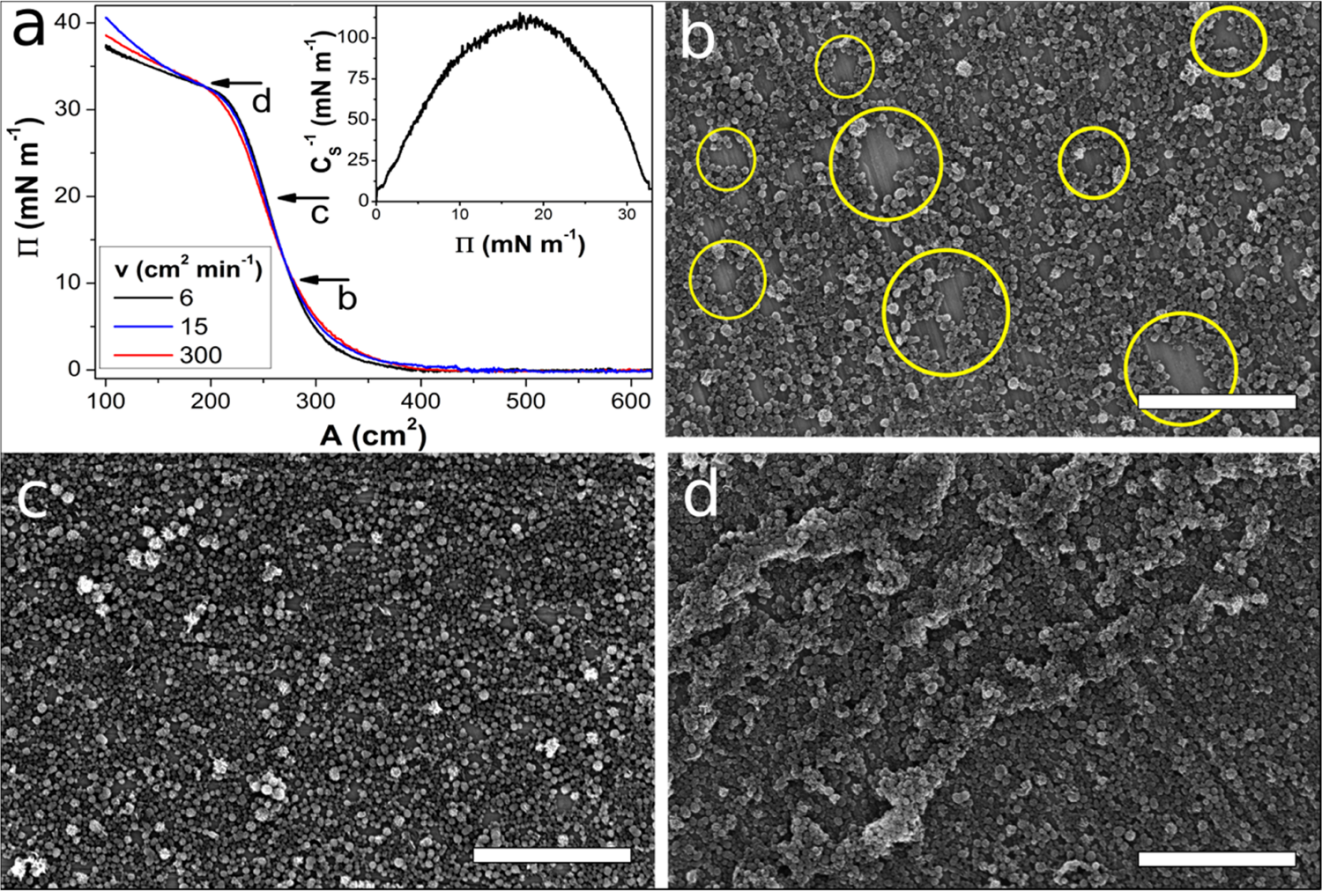
**Π**–***A*** isotherms of SWCNH floating films (a) and the corresponding compressibility module plot (inset). SEM images of SWCNH films transferred at **Π** = 10, 20, and 33 mN m^–1^ (b–d, respectively); scale bars represent 2 μm.

The SWCNH suspension in DCM was carefully spread onto the water subphase in the Langmuir PTFE trough and created a compressible floating film. In the early stages of compression, surface pressure was stable at around 0 mN m^–1^. Subsequently, **Π** starts to grow slowly with a film area (***A***) of around 400 cm^2^. During further compression, the surface pressure growth rate increased, and after the value of about 10 mN m^–1^, **Π** grew almost linearly until a collapse point at **Π** = 30 mN m^–1^ and ***A*** = 210 cm^2^. The floating film creation was independent of surface compression rate—standard **Π**–***A*** isotherms recorded for the SWCNT layers created at varied ***v*** values are shown in Figure 3a. The compression modulus (***C***_**S**_^***–*****1**^) defined as^46−49^
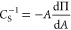
1was calculated for the floating film state analysis—the resulting plot is shown in the inset of Figure 3a. The highest ***C***_**S**_^***–*****1**^ value, about 110 mN m^–1^, was achieved for a surface pressure of about 18 mN m^–1^. The compression modulus value corresponds to a liquid condensed phase according to the criterion of Rideal and Davies.^50^ The liquid condensed phase was observed earlier for turbostratic graphene Langmuir layers,^51^ while for different nanocarbon structure floating films, significantly lower values of ***C***_**S**_^***–*****1**^ were reported.^35,52^ The relatively high value of the maximal compression modulus suggests the high density and homogeneity of the SWCNHs in the floating film. Before transferring, the floating films were relaxed—the trough area was changed to maintain specified surface pressures, and the relation between ***A*** and the initial film area (***A***_**0**_) was recorded and the results are shown in Figure S2 in the Supporting Information (SI). For the films at **Π** values lower than 30 mN m^–1^, the ***A***/***A***_**0**_ curves were smooth, and after 15 min, the value reached no lower than 0.98, which indicates stability in time and a homogeneous floating film. On the other hand, the film compressed at surface pressures equal to or higher than 30 mN m^–1^ resulted in a sharp relaxation pattern and much lower ***A***/***A***_**0**_ values after 15 min, which confirms that the floating films break after crossing the collapse point, and we can expect partially multilayer formations at **Π** higher than 30 mN m^–1^.

**Figure 4:**
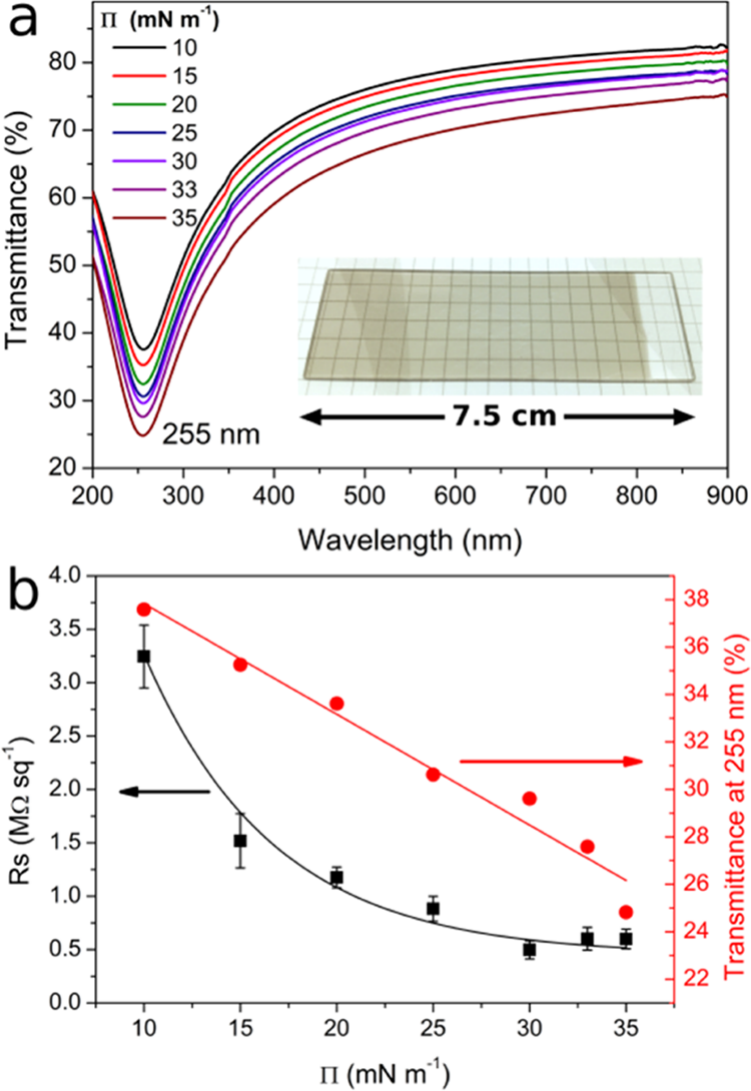
UV–vis transmittance spectra (a) and surface resistance (squares) and transmittance at 255 nm (circles) (b) of tested SWCNH films on quartz; error bars are the standard deviation of 5 measurements. The inset of (a) shows an optical image of the film transferred at 20 mN m^–1^.

In Figure 3b–d, SEM images of SWCNH layers transferred on the solid substrate at different **Π** values are shown. The film transferred at a surface pressure of 5 mN m^–1^ (Figure 3b) was inhomogeneous. Most of the layer was densely packed; nevertheless, areas of the uncovered substrate were present—the exemplary areas are marked in Figure 3b by yellow ovals. For the layers transferred at higher **Π** values, the uncovered areas were becoming rarer (see Figure S3 in the SI), and the layer compressed to **Π** = 20 mN m^–1^ was fully homogeneous. The film transferred at **Π** = 20 mN m^–1^ is not free from defects in the form of small uncovered areas and bigger agglomerates (see Figure S3 in the SI); however, we can conclude that the layer (Figure 3c) represents the closest conditions to the full coverage monolayer and its average thickness (***t***) is about the size of the individual SWCNH aggregate, i.e., 51 nm (Figure 1 inset). Low compression modulus values at the beginning of the floating film formation result from the filling of the uncovered areas and not directly from the repulsive interaction between SWCNHs. After exceeding the maximum ***C*_S_^–1^** value (layers transferred at **Π** = 25 mN m^–1^ and higher), SWCNHs start to overlap^53^ and create bigger agglomerates in the form of double- or multilayers of a few hundred nanometers in size (see Figure S3 in the SI). Furthermore, when the **Π** value exceeds 30 mN m^–1^ (collapse point in the **Π**–***A*** isotherm), the layer was damaged—most of the layer consists of multilayer agglomerates in the shape of longitudinal wrinkles (see Figure 3d). The multilayer agglomeration was also confirmed via confocal microscopy investigations. The roughness for the film transferred at 33 mN m^–1^ was much higher than that for the film obtained at **Π** = 10 mN m^–1^, which is seen in the topography representation shown in Figure S4 in the SI. Considering the above, the Langmuir method allows to control the density and arrangement of SWCNHs in the film by regulation of only one parameter—surface pressure during the layer transfer. Furthermore, the created SWCNH films were homogeneous and repeatable in a large area scale even larger than used quartz substrates with an area of almost 20 cm^2^ (see Figure 4a inset).

Additionally, knowing the concentration and volume of the SWCNH suspension used for floating film formation, we can calculate the surface density (***m***/***A***) of the transferred layer. The ***m***/***A*** values of the tested films were between 2.0 and 4.3 × 10^–6^ g cm^–2^ (see Table 1), which led to active area values of 6–12.6 cm^2^ in 1 cm^2^ of the film, estimated based on the bulk SWCNH active area. Obtained ***m***/***A*** values of the SWCNH films lead to the conclusion that the investigated films can possibly be used as an ultra-light coating and active layer modification, not affecting the weight of the final device.

**Table 1:** Basic Parameters of the Tested Layers^a^

**Π** (mN m^–1^)	10	15	20	25	30	33	35
***m***/***A*** (μg cm^–2^)	2.06 ± 0.01	2.18 ± 0.01	2.28 ± 0.01	2.40 ± 0.01	2.65 ± 0.01	3.24 ± 0.03	4.22 ± 0.10
***t*** (nm)	46.1 ± 0.8	48.9 ± 0.8	51.3 ± 0.8	55.7 ± 0.8	57.4 ± 0.8	60.9 ± 0.8	66.4 ± 0.8
***R***_**S**_ (MΩ sq^–1^)	3.244 ± 0.295	1.518 ± 0.255	1.174 ± 0.097	0.882 ± 0.117	0.497 ± 0.085	0.602 ± 0.107	0.599 ± 0.091
**ρ** (Ω·cm)	14.98 ± 1.62	7.43 ± 1.36	6.02 ± 0.59	4.91 ± 0.72	2.85 ± 0.52	3.66 ± 0.69	3.98 ± 0.65
**σ**(S cm^–1^)	0.066 ± 0.007	0.134 ± 0.025	0.166 ± 0.016	0.204 ± 0.029	0.350 ± 0.064	0.272 ± 0.052	0.251 ± 0.041
***T*** (at 550 nm)	0.777	0.767	0.752	0.737	0.731	0.716	0.686
**FOM** ×10^–3^	0.43 ± 0.04	0.87 ± 0.14	1.05 ± 0.09	1.30 ± 0.17	2.23 ± 0.38	1.72 ± 0.30	1.52 ± 0.23

a ***m***/***A***—surface density, ***t***— film thickness, ***R***_**S**_—surface resistivity, **ρ** = ***R***_**S**_ × 51 nm—electrical resistivity, **σ**—electrical conductivity, ***T***—light transmittance, **FOM**—figure of merit parameter.

### 3.3 SWCNH Film Properties

Transmittance spectra at the UV–vis region (Figure 4a) allowed us to study the electronic structure of the SWCNH films. In the visible region, the spectra are dominated by the background, smoothly decreasing with wavelength without van Hove singularity transitions.^54,55^ In the UV transmittance spectra, a strong minimum at 255 nm was found. Due to the similar structure of the SWCNHs to that of CNTs, the UV band can be attributed to the π-plasmon excitation (collective oscillations of π electrons) and originated from the intrinsic optical property of the graphite sheet.^55,56^ According to the anisotropic optical properties, graphite and other carbon allotropes are characterized by two UV bands^57^ originating from the π-plasmon excitation in directions parallel and perpendicular to the graphite c-axis. However, for the SWCNHs, only one wide band is observed,^38,58^ which is reasonable due to the isotropic SWCNH orientation in ball-shape aggregates. In general, for the π-plasmon band, the position depends on the geometries of the structure, the existence of defects, and the interactions with the environment. For SWCNHs, the UV band was often observed at about 260 nm.^59^ Agresti et al. showed the blue shift of the band with an increasing oxidation rate.^56^ This can lead to the assumption that the investigated SWCNHs are highly oxidized, as in our case, the UV band is localized at 255 nm. However, Agresti et al. studies were carried out for water suspensions of nanohorns, which makes it difficult to compare the results with our spectra recorded for the SWCNH film deposited on quartz. Furthermore, the IR transmittance spectrum does not reveal the existence of any C=O bands in the investigated SWCNHs (Figure S5 in the SI). In the IR spectrum, only two very weak bands at 1184 and 1563 cm^–1^ can be found, and they most probably originate from C=C and C–C bond vibrations, respectively.

**Figure 5:**
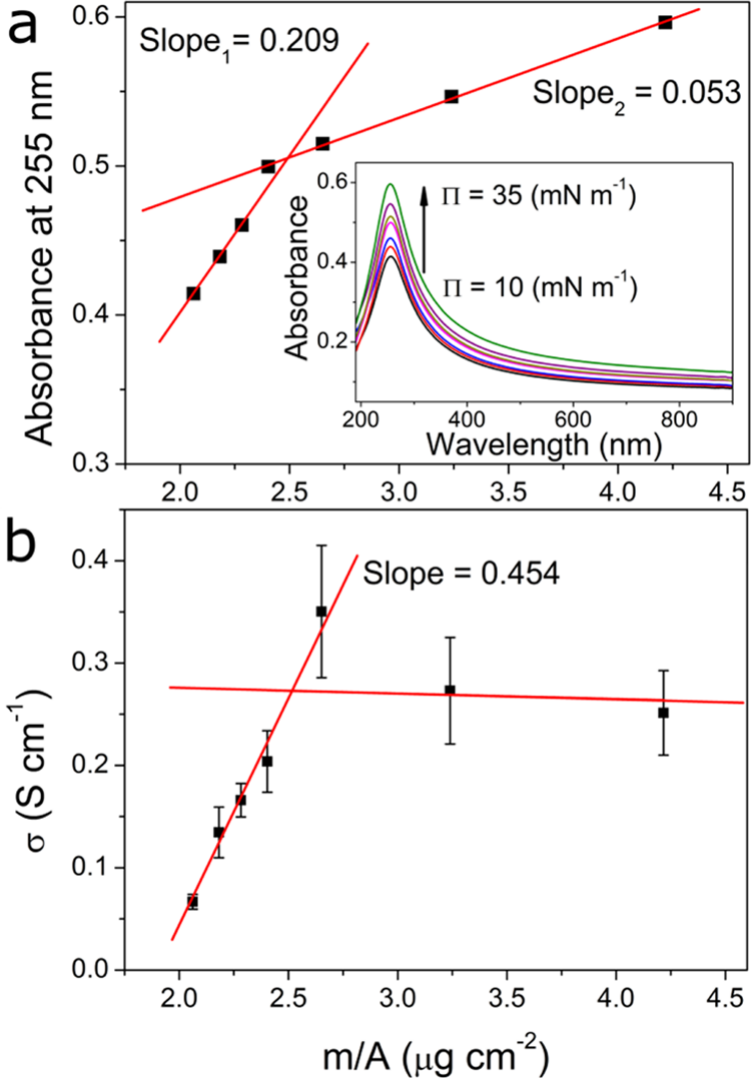
Absorbance at 255 nm (a) and conductivity (b) versus surface density of the investigated films. The inset of (a) shows the UV–vis absorbance spectra of SWCNH films transferred at different **Π** values.

The UV band position was independent of surface pressure during film transfer—derivative transmittance spectra were almost identical for every investigated film (see Figure S6 in the SI). Moreover, the transmittance value decreased linearly with **Π** (Figure 4b red circles); however, the Lambert–Beer law was not preserved for the whole investigated **Π** range. When values of absorbance at 255 nm were recorded for the films transferred at different surface pressures and plotted as a function of ***m***/***A*** (Figure 5a), two regions of significantly different slopes of linear dependency were revealed. The first region concerns the SWCNH films transferred at **Π** between 10 and 20 mN m^–1^, and the second region is for layers obtained at higher surface pressures. The reason for the change in the slope of the absorbance versus surface density relation is the presence of the agglomerated SWCNH structures in the layers of high surface pressure—the length of the optical path in the film and the absorption coefficient can differ for nanohorn mono- and multilayers. However, according to the SEM and **Π**–***A*** isotherm analysis, we assumed that the film transferred at **Π** = 20 mN m^–1^ represents conditions close to the fully packed SWCNH monolayer with an average thickness of about 51.3 nm. Furthermore, by assuming that the absorbance of the film is proportional to its average thickness, we estimate the ***t*** value for every tested film. The ***t*** values grew with surface pressure during the transfer from 46.1 to 66.4 nm. All average film thicknesses are listed in Table 1.

For investigation of electrical properties, the resistance (***R***) of the SWCNH films was recorded and recalculated to surface resistivity (***R***_**S**_), according to the following relation^26^
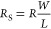
2where ***L*** and ***W*** are the tested film length and width, respectively. The ***R***_**S**_ value decreases exponentially with **Π** from 3.24 to 0.49 MΩ sq^–1^ (Figure 4b black squares), which is an expected dependence and the values typical for the carbon nanostructures monolayers. For films transferred at higher surface pressures (33 and 35 mN m^–1^), the regrowth of the ***R***_**S**_ values was observed, which comes from inhomogeneous layers—SWCNH films break after crossing the **Π**–***A*** isotherm collapse point at 30 mN/m (see Figure 3d). The resistivity (**ρ =*****R*****_S_*****t***) follows the ***R***_**S**_ values and reaches the lowest value of 2.85 Ω·cm for the film transferred at 30 mN m^–1^; the **ρ** values are shown in Table 1. The conductivity (**σ** = **ρ**^**–1**^) linearly grows with surface density at a slope of about 0.454 S·cm μg^–1^ (Figure 5b), and after reaching a maximum of about 0.3 S cm^–1^, it stabilizes.

The SWCNH film resistance decreases with the surface pressure, most probably due to the increasing number of conducting paths in the film during the growth of the surface pressure. However, increasing the density of the layer leads to a decrease in light transmittance and is unfavorable for potential applications in semi-transparent electronics. Therefore, the figure of merit (**FOM**) parameter is often calculated for the comparison of the conducting thin films simultaneously taking into account the ***R***_**S**_ and transmittance of light at the middle of the visible wavelength range. In the general form, the **FOM** is defined as^60^
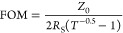
3where *Z*_0_ is the impedance of free space, equal to 377 Ω, and ***T*** is the transmittance of the film at 550 nm (the center of the visible spectrum). The **FOM** values of the investigated films were far from the industrial requirements (around 100)^61^ and varied in the range from 0.43 to 2.23 × 10^–3^ (see Table 1 and Figures S7 and S8 in the SI). Very surprising is the fact that the highest **FOM** value was obtained for the SWCNH film transferred at **Π** = 30 mN m^–1^, which is a value much higher than the surface pressure corresponding to the maximum compression modulus at about 18 mN m^–1^. This suggests the best SWCNH Langmuir–Schaefer film properties, in view of semi-transparent electrode construction, we can expect at surface pressure significantly higher than maximum ***C***_**S**_^***–*****1**^.

## 4 Conclusions

The preparation and properties of Langmuir–Schaefer films of single-wall carbon nanohorns are reported for the first time. The density and arrangement of SWCNH in the film can be controlled by the regulation of only one parameter—surface pressure during layer transfer. The transparency of the nanohorn films is comparable to those obtained for carbon nanostructure thin films, while their resistivity is much higher than those for carbon nanotube thin films, with values similar to those obtained for defective graphene nanoflake layers. Carbon nanohorn optical and electrical properties are similar to those obtained for other nanocarbon, but there are a few important differences. First of all, by using carbon nanohorns, we can avoid the introduction of metal ions, which are often present in carbon nanotubes as pollution after the production process. The specific surface area of the nanohorns is significantly larger because of their tendency to form “dahlia-like” aggregates. All these mentioned properties allow us to conclude that carbon nanohorns are very promising candidates for both model compounds that allow us to investigate complicated intermolecular interactions and scaffold photoelectrochemical sensors.

## Supporting Information Available

Raman spectra of the SWCNH powder; relaxation patterns registered for the investigated SWCNH floating films; SEM images at different magnifications of SWCNH films transferred at varied surface pressures; laser scanning microscopy (LSM) images and topography reconstructions of SWCNH films on quartz; IR transmittance spectrum of SWCNH powder dispersed in KBr pellets; UV–vis derivative transmittance spectra of investigated films and transmittance spectrum recorded for the SWCNH film transferred at 25 mN m^–1^; **FOM** versus surface pressure of investigated films; and **FOM** versus surface density of investigated films (PDF)
