# Supplementary material for: Single-Wall Carbon Nanohorn Langmuir–Schaefer Films
Source: Langmuir. 2023 Aug 16;39(34):12124–31. doi: 10.1021/acs.langmuir.3c01396 (PMC10469459; doi:10.1021/acs.langmuir.3c01396)
Supplement: Supplementary file 1 — la3c01396_si_001.pdf [file la3c01396_si_001.pdf]

Electronic supplementary information's for:

Single-wall carbon nanohorn Langmuir-Schaefer films

Kamil Kędzierski[1]*, Karol Rytel[1], Bolesław Barszcz[2], Łukasz Majchrzycki[3]

[1] Faculty of Technical Physics, Poznan University of Technology, 60-965 Poznan, Poland
[2] Institute of Molecular Physics, Polish Academy of Sciences, 60-179 Poznan, Poland
[3] Center of Advanced Technology, Adam Mickiewicz University, 61-614 Poznan, Poland
*E-mail: kamil.kedzierski@put.poznan.pl

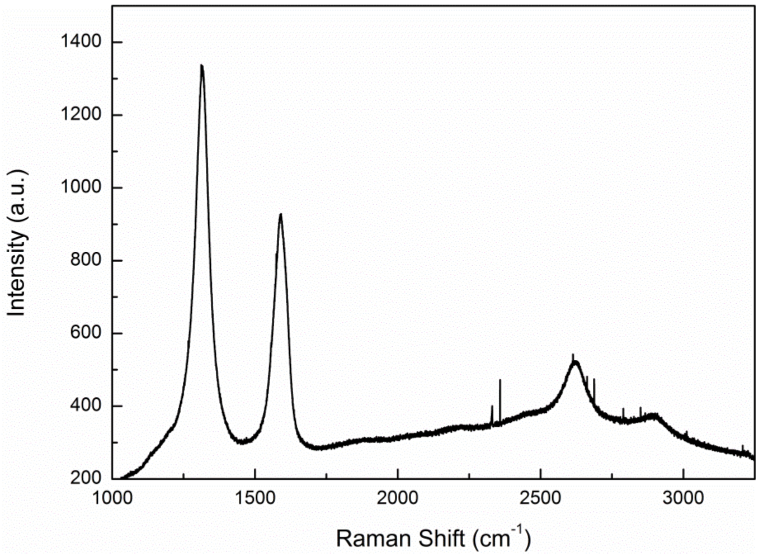

Fig. S 1 As recorded Raman spectra of the SWCNH powder.

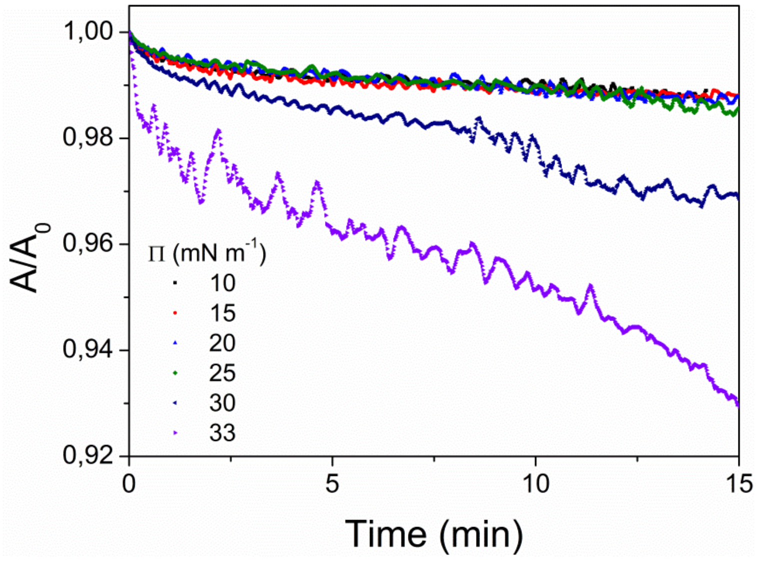

Fig. S 2 Relaxation paterns registered for the invastigated SWCNH floating films.

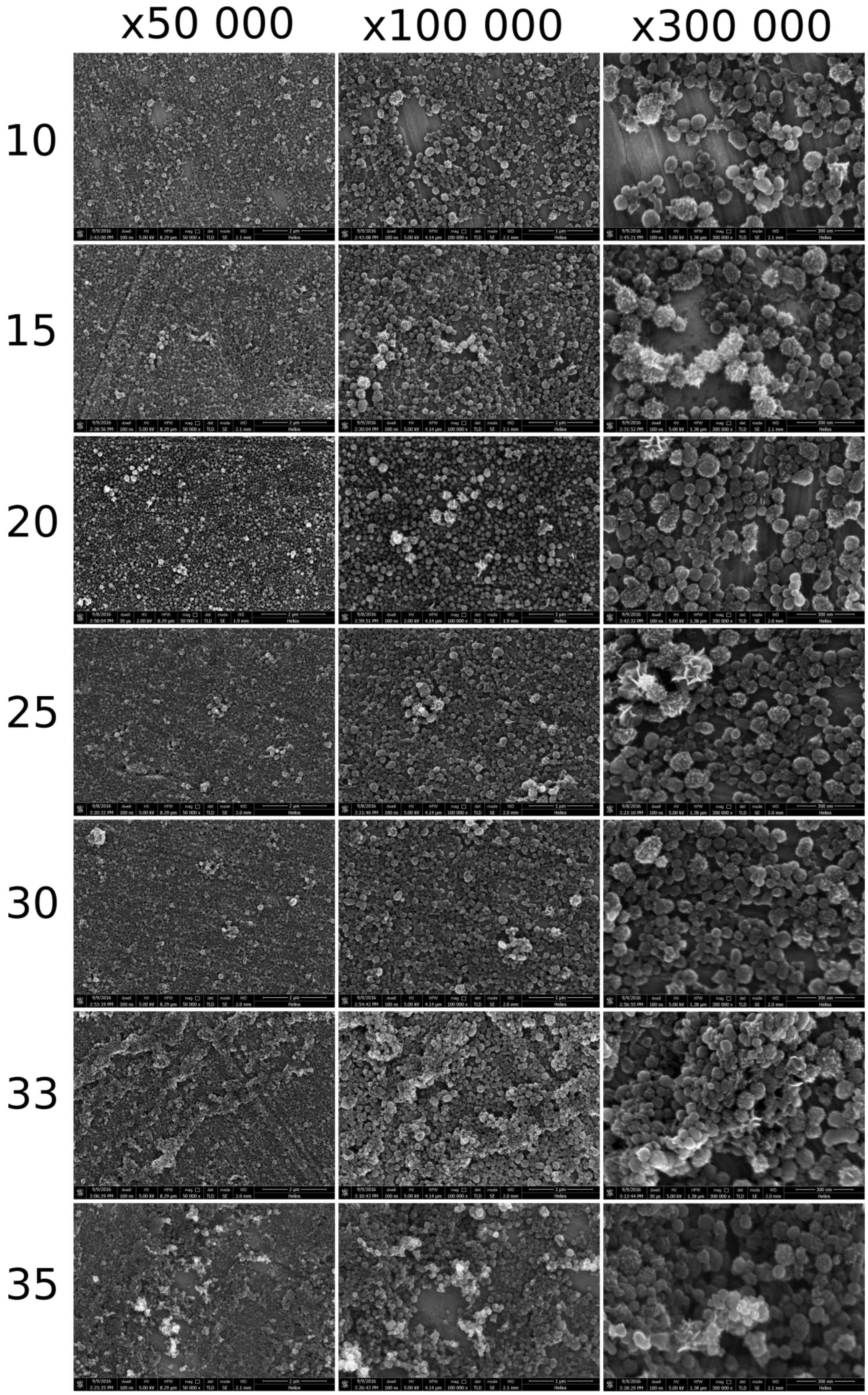

Fig. S 3 SEM images at different magnifications of SWCNH films transferred at surface pressures from 10 to 35 mN m^{-1}.

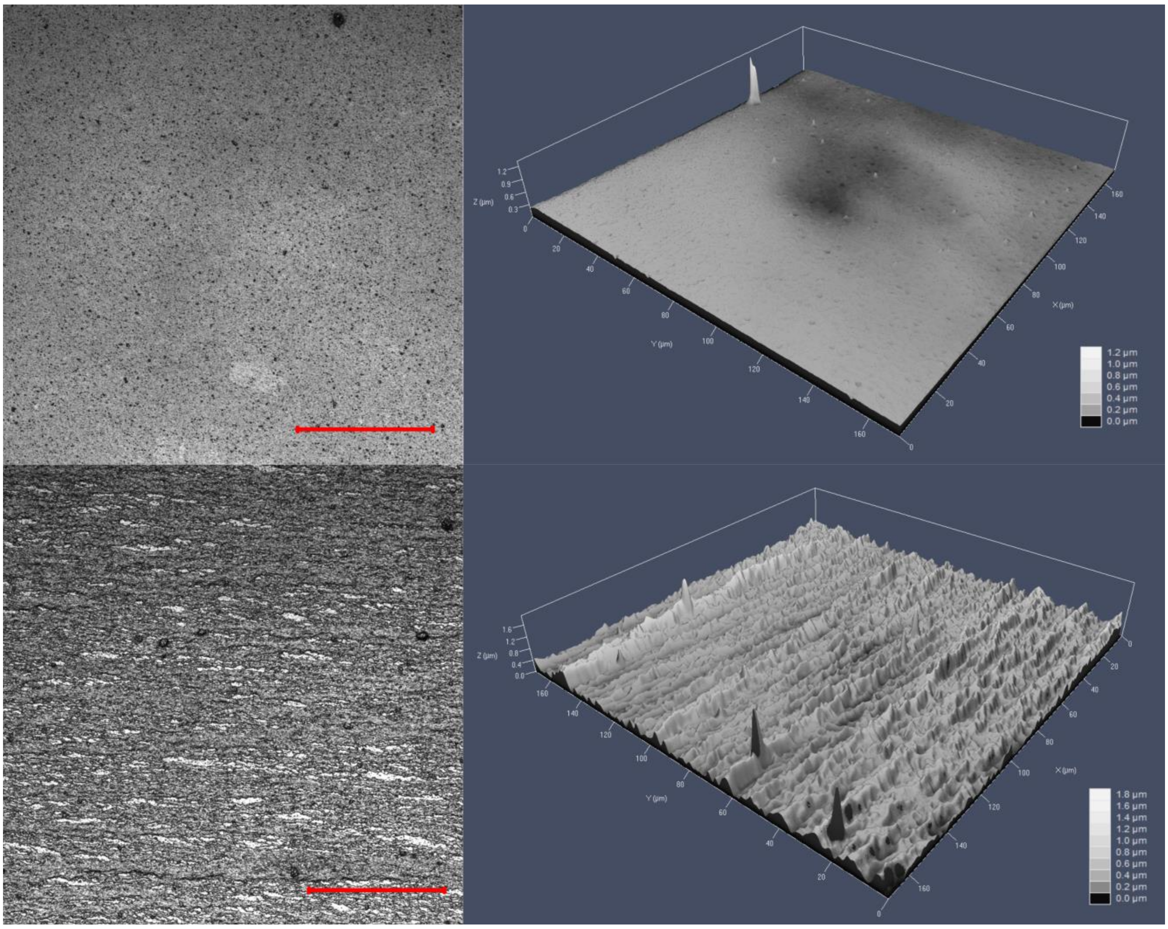

Fig. S 4 LSM images (left) and topography reconstruction (right) of SWCNH films on quartz transferred at 10 (up) and 33 (down) mN m^{-1}.

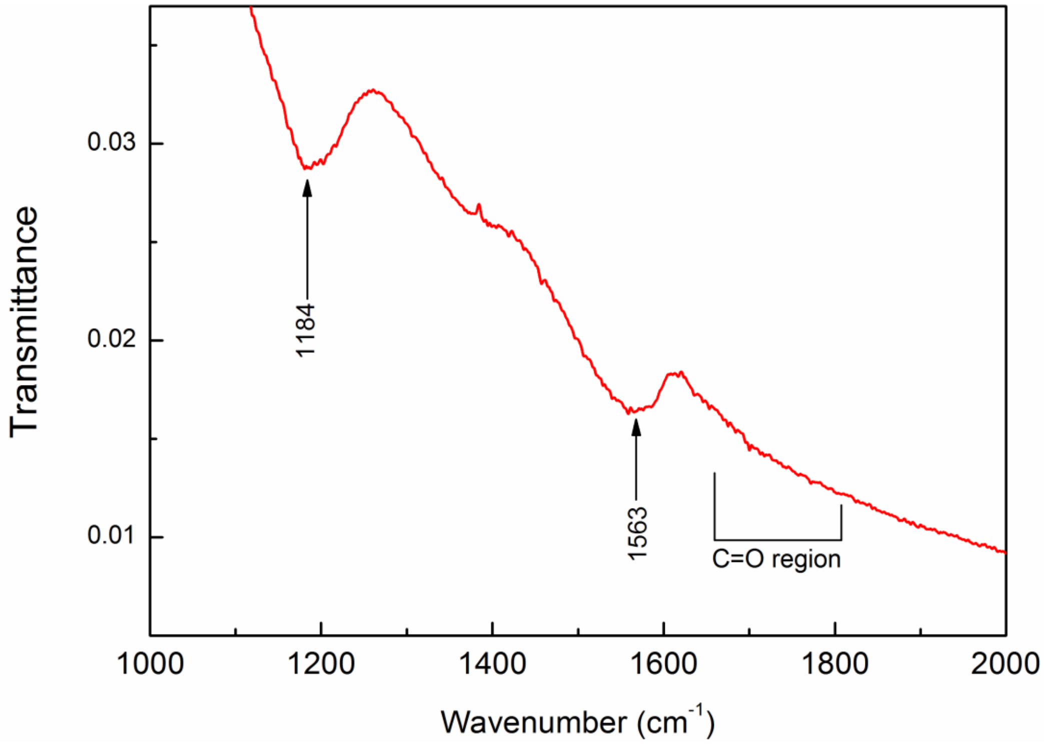

Fig. S 5 IR transmittance spectrum of SWCNH powder dispersed in KBr pellet.

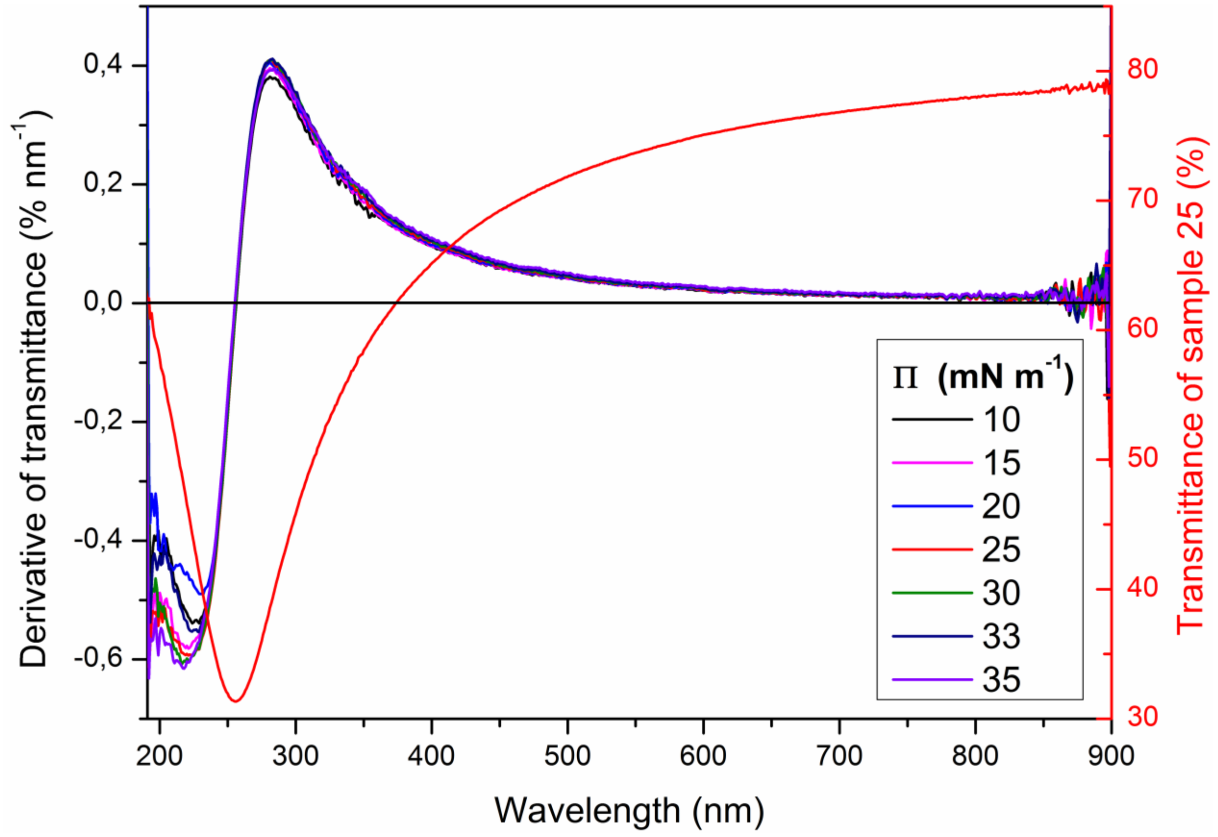

Fig. S 6 UV-Vis derivative transmittance spectra of investigated films and transmittance spectrum recorded for the SWCNH film transferred at 25 mN m^{-1} (for comparison).

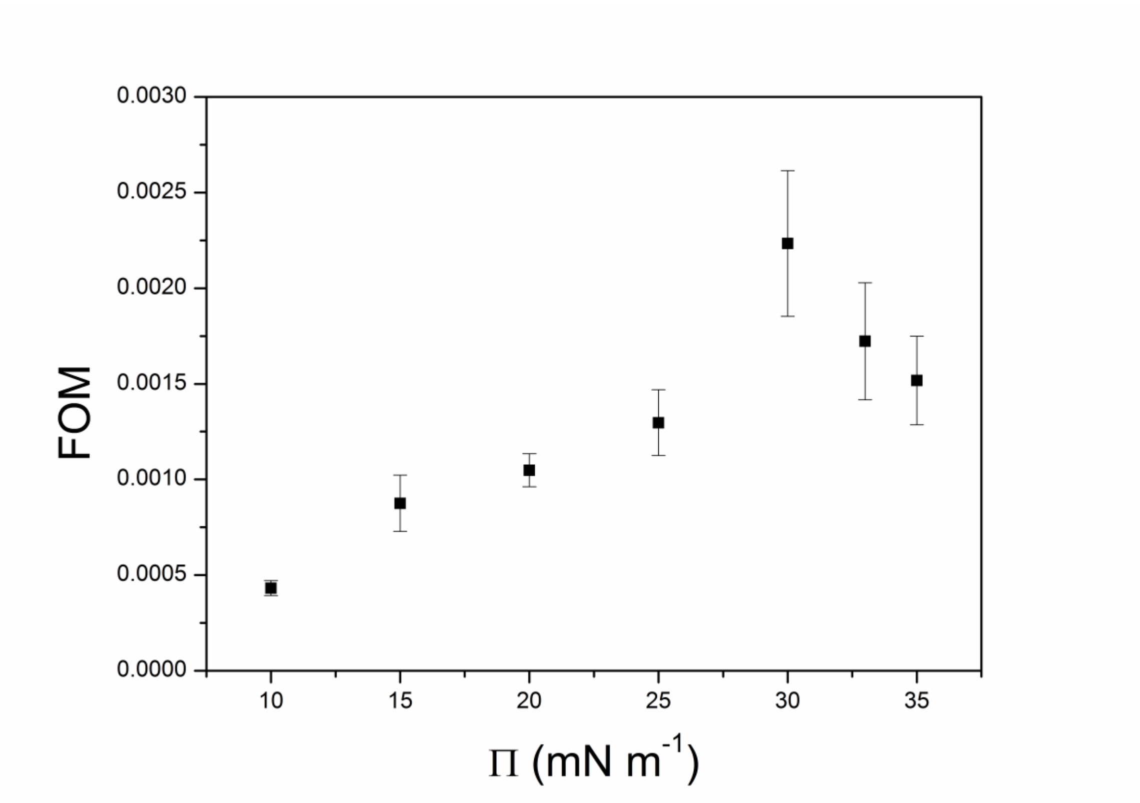

Fig. S 7 FOM versus surface pressure of investigated films

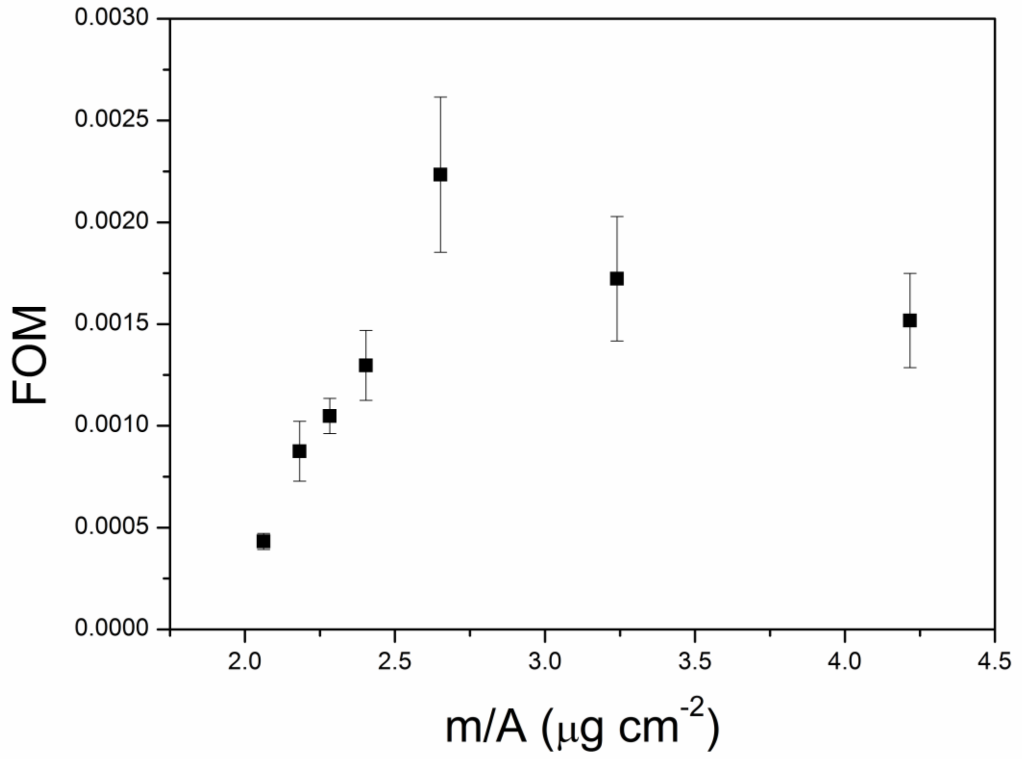

Fig. S 8 FOM versus surface density of investigated films
